# A FAS solution to a DEAD case

**DOI:** 10.1371/journal.pgen.1008842

**Published:** 2020-07-30

**Authors:** Alexandra Gruss

**Affiliations:** Université Paris-Saclay, INRAE, AgroParisTech, Micalis Institute, Jouy-en-Josas, France; French National Centre for Scientific Research, FRANCE

## CshA mutant cold sensitivity is a membrane problem

**Fig 1:**
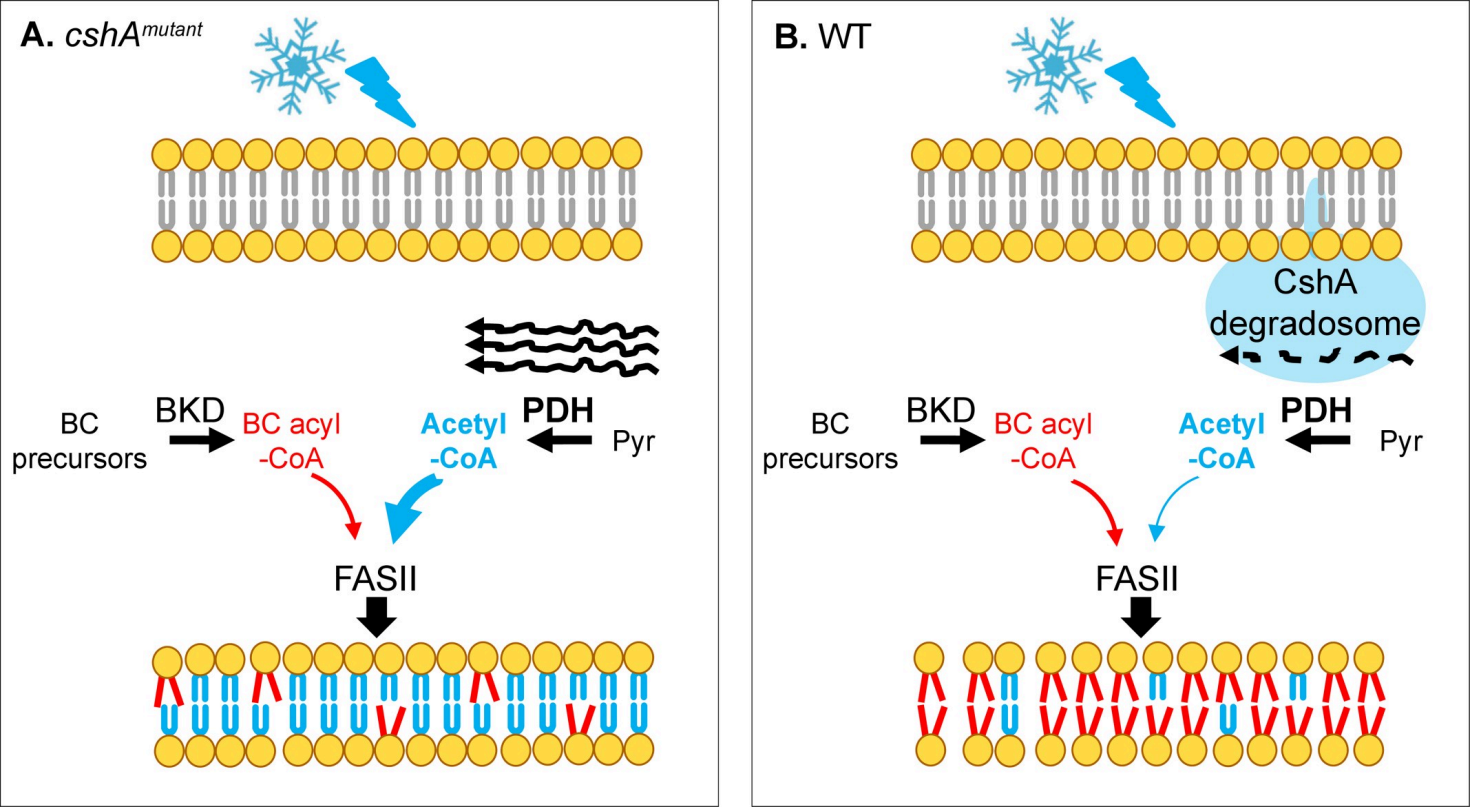
Schematic model of how CshA, the DEAD-box helicase, modifies the membrane state in cold conditions. A cold stress signal (snowflake) is transmitted via the membrane (at top), leading to membrane changes (at bottom). **(A)** *cshA* mutant. *pdh* mRNA accumulates in the cold. Greater PDH activity increases acetyl-CoA pools relative to competing branched-chain acyl-CoA substrates, favoring synthesis of straight-chain (rigid) fatty acids. Membrane rigidity accounts for cold sensitivity. **(B)** WT strain. The RNA degradosome comprising CshA is active and degrades *pdh* mRNA, which limits acetyl-CoA production. Less acetyl-CoA relative to branched-chain acyl-CoA favors BCFA synthesis, which fluidifies the membrane. Numerous *cshA* suppressor mutations restore the balance between straight and BCFA [1]. Products leading to rigid membranes are blue; those leading to fluid membranes are red. Black wavy lines represent *pdh* mRNA; dashed line indicates mRNA is degraded. BC, branched-chain; BCFA, branched-chain fatty acid; BKD, branched-chain α-keto acid dehydrogenase; PDH, pyruvate dehydrogenase; WT, wild type.

In this issue of PLoS Genetics, Khemici and colleagues make the connection between two seemingly unrelated bacterial pathways, mRNA degradation and membrane biogenesis [1]. Research of the Linder lab in Geneva has long focused on the family of DEAD-box RNA helicases and their roles in RNA and DNA metabolism [2]. Here, the focus was on the *Staphylococcus aureus* CshA helicase, which has a general role in mRNA degradation via the RNA degradosome [3]. Khemici and colleagues used cold sensitivity of the *cshA* mutant to select for cold-tolerant mutant suppressors. This simple selection led to a surprising convergence of mutant genes, which mapped nearly exclusively to membrane lipid-related functions. Remarkably, most of the 82 sequenced mutations affected the fatty acid synthesis (FASII) pathway and precursor production. The authors navigated experimentally through numerous possibilities, which led them to uncover the basis for the membrane problem in *cshA* cold-sensitive mutants. They traced the problem to a failure to degrade the mRNA of *pdh*, encoding pyruvate dehydrogenase (PDH), which in aerobic conditions produces acetyl-CoA, a hub metabolite and FASII precursor (**Fig 1**). *pdh* mRNA accumulated in the *cshA* mutant compared to wild type (WT), reasonably predicting that acetyl-CoA pools were increased. In *S*. *aureus*, FASII uses acetyl-CoA to produce straight-chain saturated fatty acids (SCFA), which rigidify membranes. But acetyl-CoA competes with branched-chain acyl-CoA, the precursors for branched-chain fatty acids (BCFA), which fluidify membranes [4]. This SCFA to BCFA ratio is critical to membrane fluidity. Fatty acid extractions showed that SCFA to BCFA ratios were elevated in the *cshA* mutant compared to the WT strain. Less PDH (or more BCFA precursor production) in tested suppressor mutants restored this ratio to that of the WT, so that the membrane would regain fluidity. This highly documented study identifies *pdh* mRNA as the essential degradosome target for cold survival, and highlights the intimate connection between membrane state and central metabolism via acetyl-CoA.

## Why are FASII genes targets for *cshA* suppressors?

Most *cshA* suppressors mapped to FASII or associated pathways. Mutations in FASII-related genes all presumably slow down phospholipid synthesis. Khemici and colleagues generalized this observation by showing that subinhibitory amounts of triclosan, which inhibits the FASII pathway protein FabI, suppressed *cshA* cold sensitivity. Their study also revealed that *fakA*, encoding a fatty acid kinase, was a *cshA* suppressor hotspot that corrected the SCFA to BCFA ratio. FakA depletion leads to free fatty acid accumulation that may modify *S*. *aureus* regulation and FASII activity and alter acetate utilization [5, 6]. How the slowdown of FASII synthesis adjusts fatty acid membrane composition and fluidity is a new open question arising from this work.

## Do all *cshA* mutant suppressors relate to the SCFA to BCFA ratio and membrane fluidity?

The selection for *cshA* cold-tolerant suppressors identified loci seemingly unrelated to membrane biogenesis. However, underlying links may exist. For example, mutations in *ndhF*, encoding a membrane protein involved in the electron transport chain, would lead to reduced respiration. Consequently higher intracellular NADH might lower PDH activity, which uses NAD as cofactor. Also, suppression by mutating RNA polymerase cofactor gene *rpoE* may have pleiotropic effects on expression of numerous membrane-related functions [7]. Mutations that impact lipoteichoic acid production and lipoprotein maturation may not directly affect phospholipid composition but could still affect membrane fluidity. Using the SCFA to BCFA ratio as readout to understand *cshA* suppressors as done here will be a valuable means of characterizing these “unknown” suppressors.

## New lines of exploration arising from the DEAD-membrane connection

The approach and findings reported by Khemici and colleagues provide a conceptual scaffold for discovering factors involved in the balance between central metabolism and the membrane. For example, anaerobic growth, which uses Pfl rather than Pdh to produce acetyl-CoA, might generate different conditions for *cshA* suppression. *S*. *aureus* incorporates exogenous unsaturated fatty acids, as enriched in host tissues [8], which might fluidify membranes and suppress *cshA* cold sensitivity, and idem for fluidifying solvents. Cold-sensitivity of cshA is suppressed in solid, but not liquid medium, raising the question of cell-to-cell contact, nutrient availability, and growth phase, in modulating membrane properties.

PDH and its product, acetyl-CoA, were identified here as the connecting link between *cshA* mutant cold survival and membrane lipid composition, as a means of adjusting membrane fluidity. Remarkably, all PDH subunits in *S*. *aureus* are prominent components of extracellular membrane vesicles [9]. Membrane blebbing could be a rapid means of removing or shuttling enzymes. Given the present study, a far-fetched but testable possibility is that PDH enrichment in extracellular vesicles might deplete intracellular PDH and favor BCFA synthesis and cell membrane fluidity. This epigenetic regulation from “without” could be an economical and rapid way to adjust membrane properties.

The membrane as a bacterial thermometer was demonstrated by several studies in the de Mendoza lab. The rigid or fluid state of membrane lipids is transmitted to membrane proteins by affecting their conformation and/or localization [10, 11]. In *S*. *aureus*, membrane fluidity affects expression of virulence factors [12]. Khemici and colleagues’ studies add CshA-degradosome turnover of *pdh* mRNA as another type of response to the membrane thermometer. The signal leading to *pdh* mRNA degradation remains to be investigated, but one possibility is that a membrane-associated degradosome component, RNase Y [13], acts as a sensor-relay of the membrane state.

The branched-chain α-keto acid dehydrogenase complex (referred to as BKD or BFM), synthesizes branched-chain acyl-CoA, the preferred competitors of acetyl-CoA in *S*. *aureus* [4]. Interestingly, BKD is a PDH complex homolog. Contrary to *pdh* mRNA degradation at low temperature, mRNA of the BKD analog in *Bacillus subtilis* is cold-stabilized [14]. It will be exciting to determine whether the *S*. *aureus* BKD complex is regulated by the degradosome under conditions where fluidity may be deleterious, e.g., at high temperature.

Altogether, this original and highly documented study brings a new clarification to the way *S*. *aureus* coordinates fundamental metabolic functions. The original conclusions and insight provided in this work will be valuable in gaining a holistic understanding of how cells respond to stress.
